# Depressive Symptoms among Chinese and Korean immigrant Family Dementia Caregivers: A Pilot Study

**DOI:** 10.1093/geroni/igaf122.2352

**Published:** 2025-12-31

**Authors:** Mina Lee, Kun Wang, Daniel Park, Jueon Kim, Ian Choi, Abigail Choi, Yeoeun Kim

**Affiliations:** Binghamton University, Binghamton, New York, United States; University of Alabama at Birmingham, Birmingham, Alabama, United States; Hackensack Meridian School of Medicine, Netley, New Jersey, United States; East Coweta Highschool, Sharpsburg, Georgia, United States; Riverside High School, Greenville, South Carolina, United States; Northview High School, Johns Creek, Georgia, United States; East Coweta Highschool, Sharpsburg, Georgia, United States

## Abstract

Taking care of family members with Alzheimer’s Disease/Alzheimer’s disease and related dementias (AD/ADRD) places a huge burden on caregivers and such a burden puts them at risk of developing depressive symptoms. Older Chinese and Korean immigrant caregivers who face cultural and language barriers may face even greater risks. Despite these challenges, little research has explored the depressive symptoms and determinants among this high-risk and underserved population. Preliminary data were collected from 42 participants (average age: 71 years; 52% female) and the study is ongoing. Online and paper-pencil surveys in English, Mandarin, and Korean were administered. Eligibility to participate are (1) self-identified as Chinese or Korean, (2) aged 50 or older, and (3) caregiving for a family member with AD/ADRD. Most reported being U.S. citizens or permanent residents, half reported being the spouse of care recipient, and 70% lived with the care recipient. More than 70% reported that they had experienced depressive symptoms at least a day the week before the survey was administered. In the preliminary analysis, depressive symptoms were significantly related to the level of caregiving burden, general health status, self-reported and cognitive decline. This pilot study highlights that many Asian American family caregivers for people with AD/ADRD suffer from depressive symptoms. As much as caring for people with dementia matters, it is also equally as important to understand and acknowledge that caregiving work takes a huge physical and emotional toll. Culturally tailored support for older dementia caregivers is necessary to prevent these family caregivers from experiencing depression.

